# Preparation, Characterization, Pharmacokinetics and Biodistribution of Baicalin-Loaded Liposome on Cerebral Ischemia-Reperfusion after i.v. Administration in Rats

**DOI:** 10.3390/molecules23071747

**Published:** 2018-07-17

**Authors:** Nan Li, Lingling Feng, Yujun Tan, Yan Xiang, Ruoqi Zhang, Ming Yang

**Affiliations:** 1 School of Pharmacy, Chengdu University of Traditional Chinese Medicine, No. 1166, Liutai Avenue, Chengdu 611137, China; nanli0724@163.com (N.L.); linglingfeng28@163.com (L.F.); P140405@163.com (Y.T.); xiangyan0325@yeah.net (Y.X.); 2 School of Pharmacy, Chengdu University of Traditional Chinese Medicine, The Ministry of Education Key Laboratory of Standardization of Chinese Herbal Medicine, Key Laboratory of Systematic Research, Development and Utilization of Chinese Medicine Resources in Sichuan Province—Key Laboratory Breeding Base of Co-founded by Sichuan Province and MOST, Chengdu 611137, China; 3 Key Laboratory of Modern Preparation of TCM, Ministry of Education, Jiangxi University of Traditional Chinese Medicine, Nanchang 330004, China

**Keywords:** baicalin, liposome, in vitro release, MCAO, pharmacokinetics, biodistribution

## Abstract

The dry root of *Scutellaria baicalensis*, has traditionally been applied in the treatment of cerebral ischemia in Chinese clinics. Baicalin (BA) is considered the key ingredient in it for the brain protection effects. The bioavailability of BA is very low because of its poor lipid and water solubility, which limits the therapeutic effects and clinical application. The aim of the present study was to develop a novel BA-loaded liposome (BA-LP) formulation to improve the drug lipophilicity and further to enhance the drug-concentration in the brain tissues. This study is also designed to investigate the pharmacokinetics of BA in the pathological conditions of stroke and evaluate the pharmacokinetic differences of BA caused by stroke after intravenous administration with BA and BA-LP. In this study, the novel BA-LP prepared in early stage were characterized by morphology, size, zeta potential, encapsulation rate and the in vitro release. The pharmacokinetics and biodistribution of BA and BA-LP were investigated by intravenous administration in rats with middle cerebral artery occlusion (MCAO) model and normal group respectively. BA-LP had a mean particle size of 160–190 nm, zeta potential of −5.7 mV, and encapsulation efficiency of 42 ± 1%. The BA-LP showed a sustained-release behavior, the in vitro drug-release kinetic model of BA-LP fit well with the biphasic dynamic model equation: Q = 1 − (60.12e^0.56t^ − 59.08e^0.0014t^). Pharmacokinetic behavior in MCAO rats is not consistent with that of normal rats. The middle cerebral artery occlusion rats got higher C_max_ and AUC_0–t_, which were about 1.5–2 times to normal rats both in BA and liposome groups. In addition, it got especially higher distribution in brain, while BA were not detected in brain tissues on normal rats. The C_max_ and AUC_0–t_ values were significantly greater with liposome than BA on both normal and MCAO rats. The tissue distribution behavior was significantly altered in the case of liposome administrated in comparison with BA, which the concentrations in the heart, liver, spleen, lungs and brain were all increased after administrated liposome, but decreased in kidneys. The TI values showed that the target of liposome was improved especially to heart, spleen and brain, and the brain’s target was higher in striatum and cerebellum. In conclusion, BA-LP might be a potential drug delivery system to improve the therapeutic efficacy of BA. In addition, these results also suggest that the pathological damages of ischemia-reperfusion have a significant impact on the pharmacokinetic traits of BA.

## 1. Introduction

In China, the dry root of *Scutellaria baicalensis* has been widely used as a traditional medicine for treating various diseases. Our ancestors had applied the herb for thousands of years. The recent studies indicated that it has protective effects against cerebral ischemia [1,2]. Baicalin (BA) is the main constituent of *Scutellaria baicalensis* [3]. The previous studies demonstrated that BA has a variety of biological activities, such as curing bacterial meningitis, and infectious brain edema [4,5], it also has the anti-tumor effect [6]. Some studies also indicated that, BA had specific radical scavenging activity and protective effects, which can protect the brain from ischemia–reperfusion injury in rats [7,8,9,10,11]. In addition, BA also has the protective effects on neurons subjecting to oxygen–glucose deprivation and reperfusion injury.

BA has been proved to exhibit so many pharmacological effects on treatment of cerebral ischemia-reperfusion injury, however, the low lipid and water solubility of BA has poor penetration of the blood–brain barrier (BBB), which is the primary obstacle to the delivery of therapeutic drugs to the brain. Thus, the clinical application of BA has been severely limited for the treatment of the brain disease [12]. Therefore, it is necessary for us to develop a novel drug delivery system to improve the solubility of BA. Liposomes, an effective nanometer-scale drug delivery system which can carry hydrophilic and lipophilic as well as amphoteric drug molecules entrapped either in the core or in the liposome bilayer [13,14]. A number of studies showed the liposome carrier could improve the solubility of drugs and change the in vivo distribution of entrapped drugs.

The objective of this study was to develop a novel baicalin-loaded liposome (BA-LP) formulation in order to improve the drug lipophilicity and further to enhance the drug-concentration in the brain tissues. The morphology, size, zeta potential, encapsulation efficiency, and the in vitro release of BA-LP have been investigated. Because the drug is used to treat diseases and only patient is the ultimate consumer of drug. It is very important to study the pharmacokinetics of drugs under the disease states. Middle cerebral artery occlusion (MCAO) is a kind of cerebrovascular disease model in rat [15]. In the pharmacokinetics and biodistribution studies, a simple, rapid, accurate and reliable method was established for determination of BA in rat plasma and tissues. Then, the pharmacokinetic characteristics of BA or BA-LP on the MCAO model have been investigated, and the experiment on the normal animal group has also been done for comparison.

## 2. Results

### 2.1. Preparation and Characterization of BA-LP

On the basis of the single factor experiments, formulation and preparation technology were optimized with encapsulation efficiency and the partial size as the indexes. 

**Figure 1:**
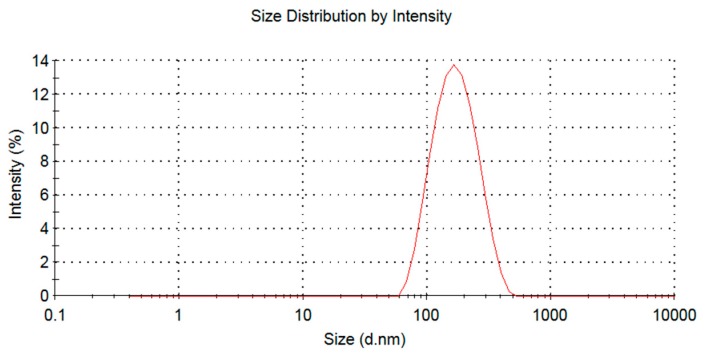
The size distribution of the BA-LP.

The optimization formulation was phospholipid and drug ratio 3.81:1, phospholipid and cholesterol ratio was 5.70:1, hydration volume was 1.02 mL, the average encapsulation efficiency of BA-LP was 42 ± 1%, the zeta potential of BA-LP was −5.7 mV, and the particle size was 160–190 nm (Figure 1).

### 2.2. In Vitro Release of BA from BA-LP

**Figure 2:**
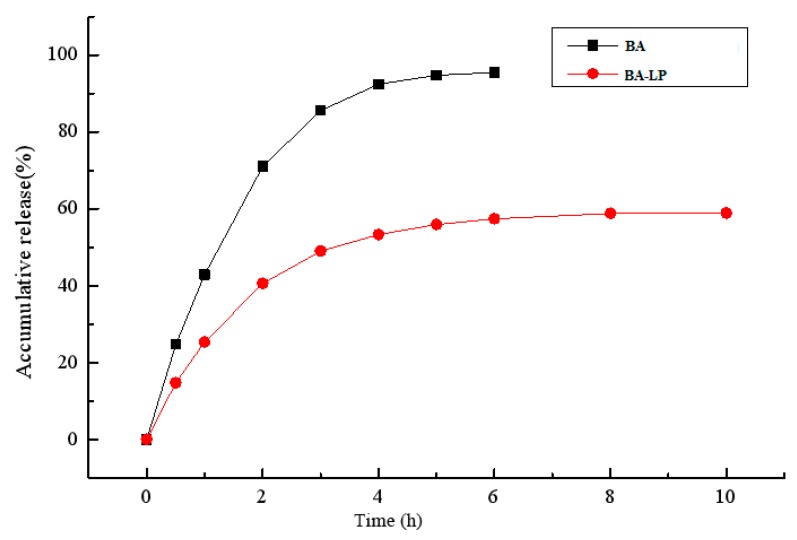
The in vitro release profile of BA solution and BA-LP.

The release of BA from the encapsulated formulation and that of the BA solution was investigated using the dialysis method. The in vitro cumulative release profile for the BA solution and BA-LP in phosphate-buffered saline (pH 7.0) are shown in Figure 2. The release of BA-LP has biphasic kinetic characteristics.

### 2.3. Pharmacokinetics Study

#### 2.3.1. HPLC Analyses

##### Selectivity

**Figure 3:**
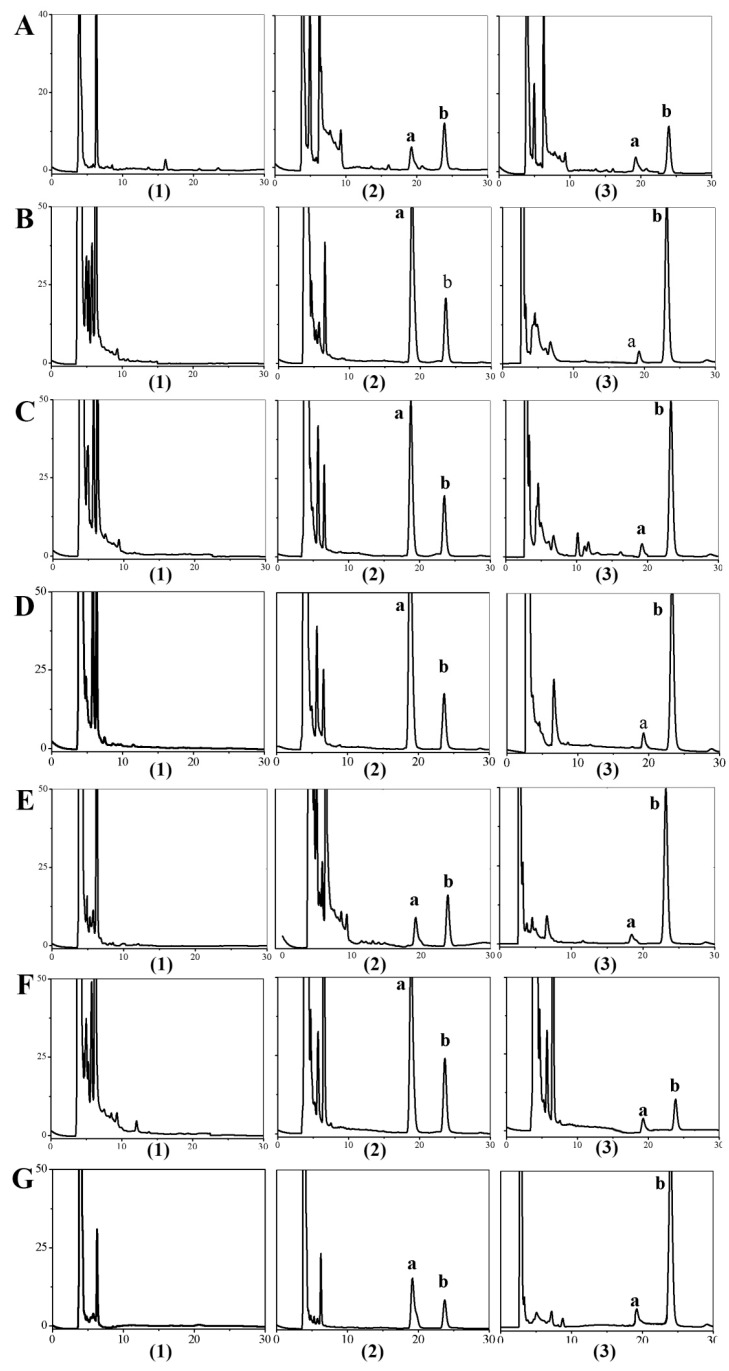
Typical chromatograms obtained from BA in samples: (**A**) Plasma; (**B**) Heart; (**C**) Liver; (**D**) Spleen; (**E**) Lung; (**F**) Kidney; (**G**) Brain; (1) blank samples; (2) samples with addition of standard reference of BA and IS; (3) samples after i.v. administration; (a) the peak of BA; (b) the peak of IS.

The selectivity of the method was obtained by comparing the blank samples before administration with the samples after addition of the BA standard and IS substance. Figure 3 shows that HPLC method had a good selectivity.

##### Linearity

As it showed in Table 1, BA in the plasma and other tissue homogenates all had good linearity within the concentration range.

##### Precision and Accuracy

The intra and inter-day precision and accuracy were showed in Table 2. The precision of BA calculated as the relative standard deviation (RSD) at various concentrations were lower than 14% for intra- and inter-day experiment. The results showed the precision and accuracy were acceptable. The accuracy of BA was higher than 89%, which means the method was acceptable.

##### Stability

The result of room temperature stability demonstrated that BA in samples could be stable at least 12 h. The result of samples’ freezing stability indicated that BA could be stored at −80 °C at least 6 days, and its accuracy was 85–99%. The result of repeating freeze stability showed that the accuracy of BA was 89–102%, which demonstrated that the samples had good stability. 

#### 2.3.2. In Vivo Pharmacokinetic Evaluation

**Figure 4:**
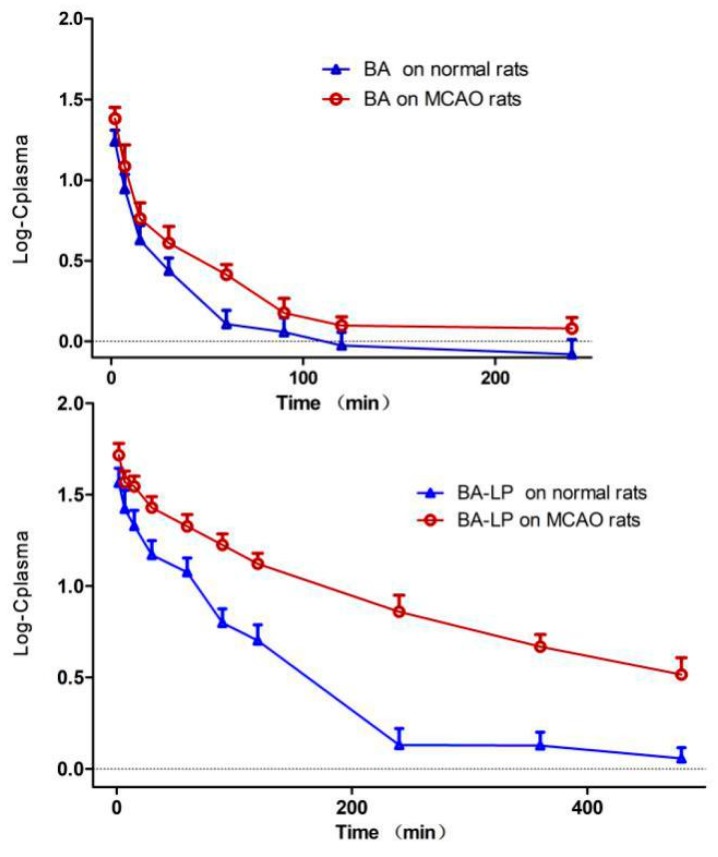
The Log plasma concentration-time profiles (mean (SD)) of BA and BA-LP on normal and MCAO animal models (*n* = 5).

The pharmacokinetic behaviors of BA in form of free drug and liposomes were investigated by using the MCAO rats and normal rats. Figure 4 depicts the Log plasma BA concentration versus time profiles in rat plasma following i.v. administration of BA and BA-LP on normal and MCAO animal model, respectively. The primary pharmacokinetic parameters of t_1/2_z, C_max_ and AUC_0–t_ are shown in Table 3.

**Figure 5:**
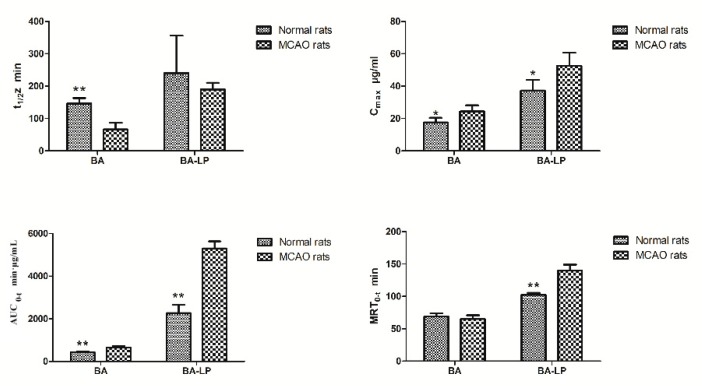
The statistical significances of t_1/2_z, C_max_, AUC_0–t_ and MRT_0–t_ between the normal and MCAO rats (*n* = 5, * p < 0.05, ** p < 0.01).

The statistical significances of t_1/2_z, C_max_, AUC_0–t_ and MRT_0–t_ between the MCAO and normal rats were shown in Figure 5. For both the BA and BA-LP groups, the C_max_ and AUC_0–t_ values were both significantly higher on the MCAO rats compared with the normal rats (24.28 (3.92) vs. 17.56 (2.84) µg/mL, *p =* 0.015; 52.48 (8.18) vs. 37.21 (6.72) µg/mL, *p =* 0.012; 652.45 (79.77) vs. 428.10 (55.04) min·µg/mL, *p =* 0.001; 5295.98 (338.67) vs. 2266.38 (383.88) min·µg/mL, *p =* 0.000), respectively. The MRT_0–t_ values with BA-LP was significantly longer on the MCAO rats compared with the normals (139.75 (9.28) vs. 102.32 (2.88) min, *p =* 0.000).

**Figure 6:**
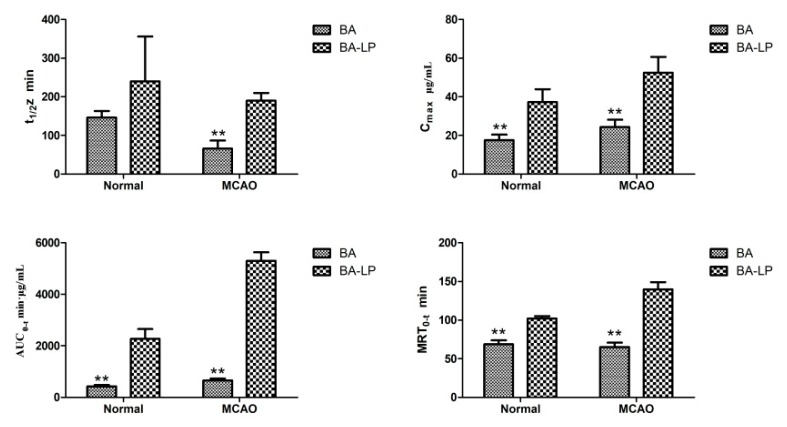
The statistical significances of t_1/2_z, C_max_, AUC_0–t_ and MRT_0–t_ between administration of BA and BA-LP (*n* = 5, ** p < 0.01).

Figure 6 depicts the statistical significances of t_1/2_z, C_max_, AUC_0–t_ and MRT_0–t_ between BA and BA-LP groups. For the normal models, the C_max_ and AUC_0–t_ values were significantly greater with BA-LP than BA (37.21 [6.72] vs. 17.56 [2.84] µg/mL, *p =* 0.000; 2266.38 [383.33] vs. 428.10 [55.04] min·µg/mL, *p =* 0.000). Similar statistical differences were found on the MCAO models (52.48 [8.18] vs. 24.28 [3.92] µg/mL, *p =* 0.000; 5295.98 [338.67] vs. 652.45 [79.77] min·µg/mL, *p =* 0.000). The MRT_0–t_ values with BA were significantly shorter compared with the BA-LP both on normal and MCOA models (68.82 [4.87] vs. 102.32 [2.88] min, *p =* 0.000; 65.15 [5.77] vs. 139.75 [9.28] min, *p =* 0.000), respectively.

#### 2.3.3. Tissue Distribution

The BA concentrations observed in the heart, liver, spleen, lung and kidney after intravenous administration with BA or BA-LP on normal and the MCAO animal model, are shown in Table 4 and Table 5. The results indicated that the concentrations of BA in the heart, liver spleen and lungs were all greatly increased after administrated BA-LP compared with BA, but decreased in kidneys.

The drug targeting parameters of Te, TI, Ce and RTE in different tissues after intravenous administration with BA or BA-LP on the MCAO models were calculated and the results are shown in Table 6.

Te represents the selective targeting to an organ, and a bigger Te value indicates that the drug delivery system is easier to target to the tissue. The BA and BA-LP were all mainly distributed in brain and kidney as shown in Table 4. Comparing with the two groups of Te values, the BA-LP showed a significant increase in the distribution of spleen, striatum and cerebellum. TI indicates the targeting on a specific organ tissue, and the TI > 1 means the preparation could target to a certain organ. TI values showed that the target of BA-LP to various tissues and organs, especially to heart, spleen and brain. The brain’s target was higher in striatum and cerebellum. RTE represents that the same drug with different preparations trends towards different tissues, and the positive RTE value indicates a significant target. So the target of BA-LP to heart, liver, spleen, striatum and cerebellum was significant, and the RTE values were 90.0, 12.8, 793.1, 49.2 and 27.6%, respectively.

**Figure 7:**
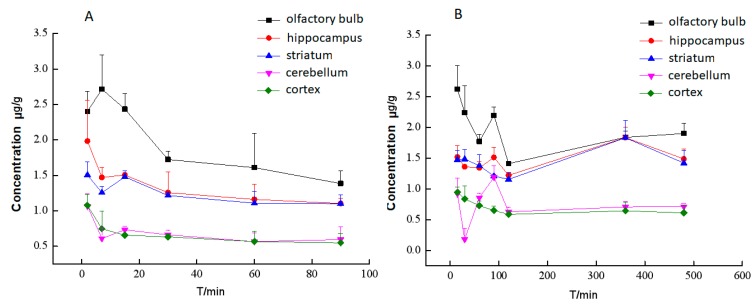
The concentrations (mean (SD)) of BA in different brain tissues after intravenous administration with BA (**A**) or BA-LP (**B**) on the MCAO model (*n* = 5).

The distribution in different brain tissues after intravenous administration with BA or BA-LP on the MCAO animal model are shown in Figure 7A,B, respectively. BA was not detected in all most of brain homogenate samples on normal rats, which indicated that the distribution to brain in rats with the MCAO model was greatly increased. Figure 7A depicts that BA could not be detected in various brain tissues 100 min after administrated BA. Comparing Figure 7A, liposomes dramatically promoted the retention time of BA in the brain.

## 3. Discussion

Since BA has been proved to exhibit so many pharmacological effects on treatment of cerebral ischemia-reperfusion injury, however, similar like most components obtained from the traditional Chinese herbs, BA possesses low lipid and water solubility, which causes low permeation into biological membranes. Therefore, improving the ability of BA to pass through the BBB is the precondition for the clinical application of BA.

Currently, liposomes have been considered as one of the most promising drug delivery carriers, it can improve drug solubility and have the potential of the targeting effect, thus to increase the therapeutic index. Therefore, in previous study, we applied the Box-Behnken design to optimize the preparation process of BA lipid system. The optimal prescription was as follows: the proportion of phospholipid drugs 3.81:1, phospholipid cholesterol ratio 5.70:1, hydration volume 1.02 mL, ultrasonic power 60 W, ultrasonic time 10 min, ultrasonic temperature 20 ± 5 °C. The resulting BA-LP was round in shape, with a particle size of 160–190 nm. The zeta potential of BA-LP was −5.7 mV, which indicated that there were a number of negative charges on the surface of BA-LP. The entrapment efficiency of BA-LP was 42 ± 1%.

The in vitro release study of BA-LP indicated that the first 4 h was an initial burst-release phase, and the in vitro accumulative release rate within 4 h was 53.35%, Therefore, the release rate became slow, demonstrating a typical sustained and prolonged drug-release behavior. The zero order kinetics, first-order kinetics, biphasic kinetics, Niebergull and Higuchi models were applied to simulate in vitro release of BA-LP. The in vitro drug-release kinetic model of BA-LP fit well with the biphasic dynamic model equation: Q = 1 − (60.12 e^0.56t^ − 59.08 e^0.0014t^) (r = 0.999). Therefore, it was speculated that the sustained -release property of BA-LP can improve the clinical therapeutic effect of BA.

Generally, most of the pharmacokinetic studies were performed on healthy animals, which could ignore the effect of pathology on pharmacokinetics. It is valuable to study the pharmacokinetic profile based on pathological animals, because the body state of animal is closer to clinic. In our study, we selected the MCAO model to simulate the state of stroke. The transient focal ischemia model is minimal harmful and has been used widely in scientific research. The pharmacokinetic study of BA in liposome form on MCAO model rats has never been reported before.

The effect of stroke on disposition and absorption of BA was investigated, the parameters between the groups of MCAO model and normal rats were compared as well. In the MCAO group, bigger values of C_max_ and AUC_0–t_ were obtained, which indicated that it may enhance the speed of distribution or reduce the elimination. This result is consistent with a previous pharmacokinetic study [15], in which, BA had a better absorption effect in the pathologic condition. These results proved the rationality of using BA in cerebrovascular disease, which would improve the therapeutic efficacy.

In the brain tissuse, the distribution of drugs in rats with the MCAO model was greatly increased both with BA and BA-LP. It suggested that stroke could increase the permeability of BBB by increasing the number of endothelial caveolae and transcytosis rate and disrupting tight junction as reported, which may enhance the transportation of drug. The vascular pathway may also play an important role in drug delivery under pathological conditions. The brain tissues are rich with microvascular. Stroke may also affect the permeability there, even when occlusion happened in middle cerebral artery [16,17].

In the pharmacokinetics study on the normal and MCAO rats, in plasma, the C_max_ and AUC_0–t_ values were significantly greater with BA-LP than BA. In addition, the other pharmacokinetic parameters were also significantly improved as evidenced by the 1.64-fold increase in the t_1/2z_ and a 4.34-fold reduction in the renal clearance rate with a 1.49-fold increase in the MRT_0–t_ on the normal animal model; a 2.87-fold increase in the t_1/2z_ and a 8.08-fold reduction in the renal clearance rate with a 2.14-fold increase in the MRT_0–t_ on the normal animal model; this improvement in the pharmacokinetic parameters could potentially lead to the prolonged the retention time of BA in vivo and thereby improve the therapeutic efficacy of BA.

The tissue distribution behavior of BA was significantly altered in the case of BA-LP administrated in comparison with BA. The concentrations of BA in the heart, liver, spleen, lungs and brain were all increased after administrated BA-LP compared with BA in both normal model and MCAO model, but decreased in kidneys. The target parameters results indicated the similar differences between BA and BA-LP. However, the mechanism of biodistribution after oral administration needs to be clarified by further studies. In addition, it’s worth mentioning that the toxicity studies of BA show that BA has very little toxicity to normal epithelial and normal peripheral blood and myeloid cells [18]. These results indicate that the liposomes for BA is helped to pass through the BBB, and may be applicable to cerebrovascular disease.

## 4. Materials and Methods

### 4.1. Materials and Reagents

BA reference substance was obtained from the National Institute for the Control of Biological and Pharmaceutical Drugs (Beijing, China), Batch No. 110715-201006; Soybean lecithin was obtained from Shanghai Ivet Medical Technology Co., Ltd. (Shanghai, China), Batch No. SY–SI-160802; Cholesterol was purchased from Shanghai Ivet Medical Technology Co., Ltd. (Shanghai, China), Batch No. B40936; Quercetin reference substance was provided by Chengdu Man Site Biotechnology Co., Ltd. (Chengdu, China), Batch No. MUST-16111114; Methanol (High Performance Liquid Chromatography grade) was from Tedia Company (Darmstadt, Germany); Double distilled water used in this study was prepared in a laboratory double distilled water purification system.

### 4.2. Preparation of BA-LP Liposomes

BA-LP was prepared by the method of reverse evaporation in our previous research, briefly, phospholipid (100 mg), cholesterol (17.5 mg) were dissolved into ethanol (18 mL). BA (25 mg) was dissolved into PBS (pH 7.0) at 5 mg/mL, and then injected the solution into the organic phase, and was disrupted in ultrasonic homogenizer for 10 min at 20 °C, after 10 min of disrupting in ultrasonic homogenizer at 20 °C, the organic solvent was evaporated on a rotary evaporator under reduced pressure to obtain the colloid. The resulting colloid was dissolved by the addition of PBS (pH 7.0) to obtain the BA-LP solution [19,20,21].

### 4.3. Physicochemical Characterization of BA-LP

The morphologies of BA-LP were examined by transmission electron microscopy (TEM). The BA-LP samples were diluted with PBS (pH 7.0) and dropped on a Formvar^®^-coated copper grid and then were air-dried for 1 min at room temperature. The particle size and zeta potential of BA-LP was measured with a Malvern Zetasizer 3000 (Malvern Instruments Ltd., Malvern, UK). The samples of BA-LP were diluted with the physiological saline (1:20) before measurement.

### 4.4. Entrapment Efficiency Study

The BA content of BA-LP was detected with HPLC. An HPLC assay was carried out by using a reverse-phase C_18_ column (XTerra^®^ MS, 4.6250 mm, 4.6 × 250 mm, Waters, Dublin, Ireland), the mobile phase was 59% double-distilled water, 41% methanol, 0.2% phosphoric acid (*v*/*v*) at a flow rate of 1 mL/min and a detection wavelength of 276 nm and the injection volume was 20 μL.

The entrapment efficiency (EE) of BA-LP was measured using the dialysis method (dialysis membrane with a molecular weight cut-off of 10,000). The BA-LP sample was placed into the dialysis membrane bag and dialysed against phosphate-bufferd saline (pH 7.4) for 7 h by gentle shaking. After dialysis, free drug (W_f_) was determined by HPLC, as established in this study. An aliquot of 0.2 mL of BA-LP was vortex-mixed with 0.8 mL of methanol for 5 min and then was filtrated through a 0.22 μm hydrophobic Millipore membrane (Tengjin Experimental Equipment Company, Tianjin, China), and the total drug contents (W_total_) was determined by HPLC. The entrapment efficiency was calculated by the following equation:

(1) $$\mathrm{Entrapment}\;\mathrm{efficiency}\;\left(\mathrm{EE}\%\right)=\frac{\left(W_{\mathrm{total}}-W_{f}\right)}{W_{\mathrm{total}}}\times 100\%$$

where EE% was the entrapment efficiency, W_f_ was the amount of free BA in the BA-LP sample, and W_total_ was the total amount of BA in BA-LP sample.

### 4.5. In Vitro Release Study

The in vitro release studies of BA-LP vs. BA solution were performed by using the dialysis bag method (molecular weight cutoff [MWCO] 10,000 Da), The PBS (pH 7.0) has been chosen as a release medium, 2 mL of BA-LP and free-BA solution was placed into dialysis bags and tightly sealed respectively. And then, the test bags were immersed in release medium at a stirring rate of 80 rpm and at 37 °C. At predetermined time points of 0.5, 1, 2, 3, 4, 6, 8, 10 h, 3 mL dissolution media was withdrawn, which was complemented with 3 mL of fresh release medium, at 37 °C, to maintain the same volume. The sample solution was centrifuged at 10,000 rpm for 10 min, and the supernatant was then injected into the HPLC system. This study was repeated three times and the result was expressed as mean ± standard deviation (SD) The in vitro release behaviors were plotted and fitted using different release dynamic models [22,23,24,25].

### 4.6. Pharmacokinetics and Tissue Distribution Studies

#### 4.6.1. HPLC Analyses

##### Chromatographic Conditions

The HPLC system (Agilent 1260, Agilent Technologies, Santa Clara, CA, USA) consisted of a XTerra^®^ MS C_18_ analytical column (4.6 mm × 150 mm, 5.0 µm, Waters, Dublin, Ireland), a pump (G1311B quaternary pump, Agilent), a UV detector and an automatic injector (G1329B, Agilent). The mobile phrase was methanol–water–phosphoric acid (41:59:0.2, *v*/*v*/*v*) at a flow rate of 1.0 mL/min with linear isocratic elution. The detector operated at 276 nm. The injection volume was 20 μL and the column temperature was 35 °C.

##### Calibration Curve

The BA reference standard and IS (meletin) were accurate weighed and dissolved in methanol, and then diluted to appropriate concentration ranges for the establishment of calibration curves in rat plasma and tissue homogenate. The concentration of stock solutions of reference substance and IS were 409.4 μg/mL and 155.28 μg/mL, respectively. The baicalin reference standard solutions at six different concentrations were prepared by spiking 300 μL blank plasma and tissue homogenate with appropriate volumes of the standard stock solution. The below were the baicalin reference standard solutions’ concentrations at plasma and tissue homogenate: the plasma were 0.11, 0.22, 5.46, 27.29, 40.94, 54.58 μg/mL, the heart were 0.054, 0.11, 1.09, 5.46, 10.92, 13.64 μg/mL, the liver were 0.054, 0.11, 1.09, 5.46, 10.92, 13.64 μg/mL, the spleen were 0.01, 0.02, 5.46, 27.29, 40.94, 54.58 μg/mL, the lung were 0.054, 0.11, 1.09, 2.73, 4.09, 5.46 μg/mL, the kidney were 0.054, 0.11, 5.46, 13.64, 21.83, 27.29 μg/mL, and the brain were 0.054, 0.11, 5.46, 13.64, 21.83, 27.29 μg/mL. BA in plasma and tissues were assayed according to a modified HPLC method. The residue was dissolved in 200 μL of methanol, centrifuged at 12,000 rpm for 10 min, and 20 μL of the solution was injected into an HPLC system [26,27].

#### 4.6.2. Animals

Sprague-Dawley rats (280–320 g) were from the Laboratory Animal Center of Jiangxi University of Traditional Chinese Medicine (Nanchang, Jiangxi, China) and were maintained at the temperature of 20 ± 2 °C on a 12-h light–dark cycle, with relative humidity of 50–60% and with free access to food and water. They were fasted for 12 h before intravenous administration. All procedures involving rats were in compliance with the ethical recommendations and guidelines for the care of laboratory animals and this animals experiment was approved by the Jiangxi University of Traditional Chinese Medicine Animal Ethical Experimentation Committee (2016KL-027).

#### 4.6.3. The MCAO Model

The MCAO model rats were induced according to the method of Longa et al. (1989) with minor modifications. Briefly, after isolating the right common carotid artery (CCA), the external carotid artery (ECA), and the internal carotid artery (ICA), the ECA and CCA were ligated, and then a 0.26-mm polylysine-coated nylon monofilament was inserted through the ICA to occlude the middle cerebral artery (MCA) in the brain, followed by a reperfusion 2 h later [28,29].

#### 4.6.4. Animal Experiment 

The rats used in this study were randomly divided into four groups with five animals at each time point. Groups 1 and 2 were used normal rats and received the intravenous BA and BA-LP at a dose of 18 mg/kg, respectively. Groups 3 and 4 were the MCAO rats with the intravenous BA and BA-LP at a dose of 18 mg/kg. After dosing for 2, 7, 15, 30, 60, 90, 120, 240, 360 and 480 min, blood was collected from the femoral artery with polyethylene pipes. Then, the blood samples were centrifuged at 1.0 × 10^4^ *g* for 10 min to separate plasma. The supernates were collected as plasma samples [30,31]. The animals were decapitated immediately after blood was collected at each time point. Then, the heart, liver, spleen, lung and kidney were isolated, and the skull was cut open and the olfactory bulb, cortex, striatum, hippocampus and cerebellum were carefully excised. After weighing, the organ samples were homogenized with normal saline in a 1:2 ratio (*w*/*w*), except striatum, hippocampus both in a 1:4 ratio (*w*/*w*) and olfactory bulb in a 1:5 ratio (*w*/*w*). All the plasma and the homogenate of each tissue were treated similar: A 300 μL aliquot of the plasma or tissue homogenates was added to 0.06 mL of 1 mol/L hydrochloric acid, 80 μL of IS working solution (155.28 μg/mL) and then mixed vigorously for 1 min. Samples were deproteinised by adding 1.2 mL of acetonitrile, followed by vigorous mixing for 4 min and centrifugation at 1.0 × 10^4^ *g* for 10 min. Then, the supernatants were evaporated to dryness under a stream of nitrogen at 35 °C and stored for up to 24 h in a freezer (−80 °C) until HPLC analysis.

#### 4.6.5. Data Analysis

In vivo pharmacokinetic evaluation: All data obtained were subsequently calculated using the software program Phoenix WinNonlin 6.3 (Pharsight Corporation, Mountain View, CA, USA). A non-compartmental model was chosen to calculate the pharmacokinetic parameters of half-life (t_1/2_z), area under the concentration-time curve (AUC_0–t_), apparent volume of distribution (Vz), peak concentration (C_max_), the mean residence time, clearance rate (CLz), etc. The statistical significances of t_1/2_z, C_max_, AUC_0–t_ and MRT_0–t_ between the MCAO and normal rats and between BA and BA-LP groups were evaluated using the *t* test, and a value of *p* < 0.05 was considered statistically significant.

## 5. Conclusions

A novel BA-LP formulation was successfully developed, the BA-LP showed a sustained-release behavior that fit well with the biphasic dynamic model equation. Compared with BA, the liposomes for BA is helped to prolong the retention time of the drug in vivo, and also to increase the drug-concentration in the brain, which may be applicable to cerebrovascular disease. The BA-LP might be a potential drug delivery system to improve the therapeutic efficacy of BA. Comparative pharmacokinetics of BA on a normal and MCAO model indicated that a pathological state increased drug-concentration in the brain, more drug may pass into brain through the vascular pathway. The result of this study would help to guide the clinical application of BA in the patients with stroke.

## Figures and Tables

**Table 1:** The standard curve of BA in plasma and tissues.

Samples	Regression Equation	r	Range (μg/mL)
Plasma	Y = 0.2115X − 0.1743	0.999	0.11–54.58
Heart	Y = 0.0443X + 0.0052	0.999	0.054–13.64
Liver	Y = 0.0315X + 0.0017	0.999	0.054–13.64
Spleen	Y = 0.0824X + 0.006	0.999	0.011–54.58
Lung	Y = 0.0973X − 0.0016	0.999	0.054–5.46
Kidney	Y = 0.05X − 0.0114	0.999	0.054–27.29
Brain	Y = 0.1117X − 0.0388	0.999	0.054–27.29

**Table 2:** Precision and Recovery of the baicalin in plasma and tissues in rat.

Samples	Concentration (μg/mL)	Intra-Day (*n* = 5)		Inter-Day (*n* = 3)	
		RSD (%)	Accuracy (%)	RSD (%)	Accuracy (%)
Plasma	40.94	5.31	89.76 ± 5.31	7.30	91.56 ± 8.22
	27.29	6.98	89.57 ± 6.97	7.69	91.56 ± 8.22
	0.22	7.06	89.72 ± 7.06	6.47	88.67 ± 6.43
Heart	21.83	7.50	92.08 ± 7.51	6.76	96.89 ± 11.23
	13.64	7.41	92.12 ± 7.41	6.73	95.02 ± 11.06
	0.11	7.19	89.44 ± 7.19	7.77	91.56 ± 8.22
Liver	10.92	6.99	92.18 ± 6.99	6.50	87.99 ± 6.75
	5.46	6.49	91.42 ± 6.48	6.80	94.04 ± 11.03
	0.11	7.21	90.27 ± 7.21	7.84	90.59 ± 8.79
Spleen	40.94	8.27	89.26 ± 7.21	5.52	93.63 ± 8.77
	27.29	4.47	89.17 ± 7.21	3.51	91.35 ± 3.78
	0.02	8.71	91.19 ± 5.72	9.17	92.01 ± 8.20
Lung	4.09	5.83	91.13 ± 5.83	5.52	93.63 ± 8.77
	2.73	4.72	91.09 ± 4.72	3.51	91.35 ± 3.78
	0.11	6.69	89.91 ± 6.69	8.05	89.99 ± 9.12
Kidney	21.83	7.73	91.74 ± 7.72	8.19	89.98 ± 9.07
	13.64	7.85	89.96 ± 7.85	8.32	89.98 ± 9.07
	0.11	9.98	89.54 ± 9.98	9.62	93.92 ± 9.01
Brain	21.83	5.55	90.24 ± 5.55	6.42	94.60 ± 11.30
	13.64	8.29	91.23 ± 8.29	7.47	86.79 ± 7.43
	0.11	7.25	90.09 ± 7.25	6.75	87.74 ± 6.96

**Table 3:** Pharmacokinetic parameters of BA and BA-LP on normal and MCAO animal models (*n* = 5). Values are mean (SD).

	BA		BA-LP	
	Normal Rats	MCAO Rats	Normal Rats	MCAO Rats
t_1/2_z, min	146.01 (16.99)	66.02 (20.87)	239.92 (116.18)	189.98 (20.00)
C_max_, µg/mL	17.56 (2.84)	24.28 (3.92)	37.21 (6.72)	52.48 (8.18)
AUC_0–t_, min·g/mL	428.10 (55.04)	652.45 (79.77)	2266.38 (383.88)	5295.98 (338.67)
AUC_0–∞_, min·µg/mL	607.50 (93.26)	769.55 (62.83)	2676.03 (556.03)	6216.73 (507.66)
Vz, mL/kg	6311.81 (794.22)	2260.37 (814.52)	2286.06 (698.53)	796.09 (88.72)
Cl, mL/(min·kg)	30.26 (5.11)	23.51 (1.88)	6.96 (1.39)	2.91 (0.25)
MRT_0–t_, min	68.82 (4.87)	65.15 (5.77)	102.32 (2.88)	139.75 (9.28)
MRT_0–∞_, min	180.78 (26.28)	107.71 (24.22)	216.25 (75.92)	230.39 (26.98)

The plasma samples were obtained with five rats at each time point.

**Table 4:** The concentrations of BA in different tissues on normal models after i.v. administrated BA and BA-LP (*n* = 5). Values are mean (SD).

Time (min)	Concentration in Tissues (μg/g)									
	Heart		Liver		Spleen		Lung		Kidney	
	BA	BA-LP	BA	BA-LP	BA	BA-LP	BA	BA-LP	BA	BA-LP
15	0.34 (0.14)	0.74 (0.39)	1.06 (0.16)	1.76 (0.18)	0.36 (0.04)	2.52 (1.90)	1.09 (0.20)	2.36 (0.47)	15.01 (1.44)	6.51 (1.29)
30	1.29 (0.78)	1.95 (0.32)	3.18 (0.34)	8.75 (2.18)	0.32 (0.04)	3.71 (1.04)	0.96 (0.20)	1.50 (0.54)	6.72 (1.71)	7.10 (2.18)
60	0.36 (0.10)	4.07 (0.45)	0.48 (0.16)	7.53 (1.93)	0.15 (0.02)	8.84 (2.59)	0.41 (0.09)	2.92 (0.54)	2.52 (0.44)	6.77 (1.62)
90	0.20 (0.08)	4.44 (0.81)	0.27 (0.18)	2.21 (0.86)	0.06 (0.01)	1.44 (0.78)	0.23 (0.03)	0.76 (0.18)	1.84 (0.32)	6.49 (0.65)
120	0.10 (0.02)	0.68 (0.34)	0.06 (0.01)	0.99 (0.31)	0.02 (0.004)	0.93 (0.33)	0.12 (0.02)	0.36 (0.17)	1.25 (0.20)	2.21 (0.50)
240	ND	0.43 (0.06)	ND	1.05 (0.30)	ND	0.20 (0.06)	ND	0.18 (0.05)	ND	0.94 (0.12)
360	ND	0.11 (0.04)	ND	0.35 (0.18)	ND	0.11 (0.16)	ND	0.16 (0.10)	ND	0.74 (0.15)

The tissue samples were obtained with five rats at each time point. ND means not detected.

**Table 5:** The concentrations of BA in different tissues on MCAO models after i.v. administrated BA and BA-LP (*n* = 5). Values are mean (SD).

Time (min)	Concentration in Tissues (μg/g)									
	Heart		Liver		Spleen		Lung		Kidney	
	BA	BA-LP	BA	BA-LP	BA	BA-LP	BA	BA-LP	BA	BA-LP
15	1.15 (0.12)	1.92 (0.28)	2.74 (0.64)	5.49 (0.72)	0.29 (0.05)	3.49 (0.34)	2.92 (0.21)	4.37 (0.36)	11.20 (1.09)	8.45 (1.20)
30	0.57 (0.07)	1.22 (0.28)	1.82 (0.16)	4.26 (0.26)	0.13 (0.01)	2.43 (0.18)	2.24 (0.21)	3.04 (0.24)	6.02 (0.29)	4.91 (0.43)
60	0.32 (0.03)	1.81 (0.30)	1.08 (0.14)	3.78 (0.62)	0.06 (0.02)	6.03 (0.38)	0.59 (0.12)	2.03 (0.35)	2.71 (0.40)	4.33 (0.53)
90	0.14 (0.03)	1.54 (0.09)	0.22 (0.05)	3.25 (0.43)	0.05 (0.02)	3.26 (0.15)	0.33 (0.03)	1.56 (0.11)	0.97 (0.26)	5.04 (0.59)
120	0.08 (0.03)	0.96 (0.26)	0.10 (0.01)	2.64 (0.25)	0.01 (0.01)	1.54 (0.12)	0.09 (0.01)	1.21 (0.22)	0.27 (0.05)	1.93 (0.17)
240	ND	1.21 (0.32)	ND	1.56 (0.28)	ND	1.28 (0.13)	ND	1.18 (0.10)	ND	2.46 (0.41)
360	ND	0.76 (0.14)	ND	1.27 (0.20)	ND	0.86 (0.23)	ND	0.69 (0.09)	ND	1.17 (0.14)
480	ND	0.40 (0.14)	ND	0.79 (0.21)	ND	0.66 (0.17)	ND	0.50 (0.15)	ND	0.90 (0.28)

The tissue samples were obtained with five rats at each time point. ND means not detected.

**Table 6:** The targeting parameters after intravenous administration with BA or BA-LP on the MCAO animal models.

Tissue	Te_(__BA)_ (%)	Te_(__BA-LP)_ (%)	TI	Ce	RTE (%)
Heart	2.4	4.6	13.8	1.8	90.0
Liver	5.7	6.4	8.2	2.0	12.8
Spleen	0.6	5.7	64.7	20.9	793.1
Lung	5.5	4.2	5.5	1.5	−23.9
Kidney	21.0	9.2	3.2	0.8	−55.9
Olfactory bulb	15.7	15.2	7.0	1.2	−3.2
Hippocampus	14.2	12.4	6.3	1.0	−13.1
Striatum	17.8	26.6	10.8	1.3	49.2
Cerebellum	5.6	7.2	9.2	1.7	27.6
Cortex	11.4	8.6	5.5	1.4	−24.7

The tissue samples were obtained with five rats at each time point.
